# Improved Thermal Insulation and Mechanical Strength of Styrene-Butadiene Rubber through the Combination of Filled Silica Aerogels and Modified Glass Fiber

**DOI:** 10.3390/ma16175947

**Published:** 2023-08-30

**Authors:** Guofeng Wang, Wenwen Yu, Sitong Zhang, Kaijie Yang, Wenying Liu, Jiaqi Wang, Fuyong Liu

**Affiliations:** College of Materials Science & Engineering, Taiyuan University of Technology, Taiyuan 030024, China; wangguofeng0259@link.tyut.edu.cn (G.W.); zhangsitong0356@link.tyut.edu.cn (S.Z.); yangkaijie0253@link.tyut.edu.cn (K.Y.); liuwenying0037@link.tyut.edu.cn (W.L.); wangjiaqi0223@link.tyut.edu.cn (J.W.); liufuyong@tyut.edu.cn (F.L.)

**Keywords:** styrene-butadiene rubber, silica aerogel, glass fiber, thermal conductivity, mechanical properties, rheological behavior

## Abstract

To improve heat dissipation capability and enhance mechanical properties, a series of silica aerogel (SA) and modified glass fiber (GF)-filled SBR composites were prepared. It was found that the addition of SA successfully reduced the thermal conductivity of SBR by 35%, owing to the heat shield of the nanoscale porous structure of SA. Moreover, the addition of modified glass fiber (MGF) yielded a significant increase in the tensile and tear strength of SBR/SA composite rubber of 37% and 15%, respectively. This enhancement was more pronounced than the improvement observed with unmodified GF, and was attributed to the improved dispersion of fillers and crosslinking density of the SBR matrix. Rheological analysis revealed that the addition of SA and MGF weakened the *ω* dependence. This was due to the partial relaxation of immobilized rubber chains and limited relaxation of rubber chains adsorbed on the MGF. Furthermore, the strain amplification effect of MGF was stronger than that of GF, leading to a more pronounced reinforcing effect.

## 1. Introduction

Styrene-butadiene rubber (SBR) [1,2,3,4] is the largest variety of general-purpose synthetic rubber. It is a random copolymer of butadiene and styrene, and its processing and product performance are similar to those of natural rubber. SBR is widely used due to its good heat resistance, aging resistance, and other desirable properties. In recent years, advancements in science and technology have led to strict requirements for the thermal conductive materials of certain SBR rubber products, such as rubber conveyor belts for carrying hot materials, tires [4], and seals used in thermal insulation environments. Most SBR rubber compounds are designed to increase thermal conductivity and improve heat transfer [5,6,7]. This helps to transfer the heat inside the compound in a timely manner, which inhibits the process of thermal aging and thermal degradation. However, increasing thermal conductivity can have adverse effects on some sealing rings and seals used in high-temperature thermal insulation environments. This is because as the external temperature rises, increasing thermal conductivity will significantly catalyze the aging of SBR rubber products. The heat causes the SBR rubber to expand, which in turn improves the diffusion rate of oxygen and activates the reaction between oxygen and rubber, resulting in thermal oxygen aging. Thus, there is a need to find a balance between thermal conductivity and the prevention of thermal aging for such rubber products. The design of SBR materials with tutored heat-dissipation capability to prevent degradation holds great promise for cost-effective and efficient industrial applications.

Aerogel-filled composites are known for their outstanding thermal insulation and mechanical strength [8]. This is due to the fact that aerogels [9] are nano porous solid materials with a continuous three-dimensional network structure, offering a specific surface area that can range from 80% to 99.8% and can reach over 1000 m^2^/g, while having a density as low as 3 kg/m^3^ [10]. The unique porous structure of aerogels consists of nanoscale pores with an “infinite” number of pore walls, each functioning as a heat shield [11]. This feature produces an effect close to that of an “infinite number of heat shields,” making aerogels the solid material with the smallest density and the best thermal insulation performance currently available. SiO_2_ aerogels (SA), which are highly transparent, have good thermal insulation properties, a low dielectric constant, and high temperature resistance, are widely used in many fields [12,13,14,15].

To further improve the mechanical properties of composite materials, fiber reinforcing fillers can be added after adding silica, aerogel and other reinforcing fillers. Chopped glass fiber (GF) [16,17] has heat resistance, high tensile strength, good insulation, anti-corrosion and good chemical stability. Due to the excellent properties of GF, it is widely used in various fields [18,19,20]. However, unmodified GF has a smooth surface and few active groups and is inert [21]. When compounded with styrene-butadiene rubber, the fibers are debonded and pulled from the matrix, resulting in poor mechanical properties. To improve the overall comprehensive performance of the composite material and reflect the value of adding fiber-reinforced fillers, we used the silane coupling agent Si-69 bis-[*γ*-(triethoxysilyl)propyl] tetrasulfide (TESPT) to modify the GF surface. The present study investigates the influence of SA-and TESPT-modified GF on thermal conductive, rheological and mechanical properties of SBR composites. The TESPT may part in interactions with SBR and GF fillers, facilitating GF dispersion in SBR composites for improved mechanical properties and influencing the nonlinear Payne effect, which gives insight into the microstructural behavior of composites.

## 2. Materials and Methods

### 2.1. Materials

SBR, brand 1712, of which the styrene content is 23.5%, oil content is 27.3%, Mooney viscosity ML (1 ± 4) 100 °C is 51, produced by Sinopec Qilu Petrochemical Company (Zibo, China); Zinc oxide, produced by Liuzhou Zinc Products Company (Liuzhou, China); Stearic acid, produced by Fengyi Oil Technology Co., Ltd. (Tianjin, China); Accelerator DM (MBTS), produced by Tianjin Organic Chemical Plant; Antioxidant 4010, produced by Shandong Shangdian Chemical Co., Ltd. (Heze, China). The specific surface area of silica (nano-SiO_2_) is 200 m^2^/g, the particle size is 12 nm, and it is produced by the Wacker Company of Germany (Munich, Germany). Sulfur (S-80), produced by Shandong Shangshun Chemical Company (Heze, China). The density of SiO_2_ aerogel (SA) is only 3.55 kg/m^3^, only 2.75 times the air density, the porosity can be as high as 99.8%, and the surface organic modification grafting rate is 70%; it is produced by Shanxi Yangzhong New Material Co., Ltd. (Shanxi, China). Chopped glass fiber (GF), 6 mm long, 10 diameter μm. Commercially available Silane coupling agent Si-69 bis-[*γ*-(triethoxysilicon)propyl] tetrasulfide (TESPT), produced by Shanghai McLean Biochemical Technology Co., Ltd. (Shanghai, China).

### 2.2. Surface Modification of GF

First, the surface of the glass fiber (GF) was cleaned with 80% anhydrous ethanol solution. The cleaned and dried GF was then immersed in a 10% TESPT ethanol solution and mechanically stirred for 30 min. After that, it was washed 6~9 times with 80% anhydrous ethanol and dried to obtain modified glass fiber (MGF).

### 2.3. Preparation of GF/SA Filled SBR Composites

SBR was plasticized on a double-roll mill (BP-8175-AL, Baopin Precision Instrument, Dongguan, China) and then placed into an internal mixer (BP-8172-B, Baopin Precision Instrument, China). Zinc oxide (5 g), fatty acid (2 g), TESPT (6 g), MBTS (1 g), antioxidant 4010 (1.5 g), nano-SiO_2_ (10 g), and S-80 (1.5 g) were used to prepare a blank control sample, which is recorded as SBR. On this basis, SA and GF were added and mixed, and the additives were added in, according to Table 1. The composite compound obtained by adding different amounts of SA was recorded as SBR/SA. Once the various fillers were evenly dispersed, GF and MGF were mixed to obtain composite materials, which were recorded as SBR/SA/GF and SBR/SA/MGF. The mixture was then discharged, and the fibers were oriented and unpacked on an open mill and parked for use. The rubber compound was then vulcanized on a flat vulcanizer (BP-8170-A, Baopin Precision Instrument, China) based on the test results of the rotorless vulcanizer (MDR 3000 Basic, MonTech, Buchen, Germany). During the vulcanization process, the rubber material was subjected to a pressure of 10 MPa at 160 °C. After the vulcanization process was completed, the vulcanizate was parked or left to rest for 24 h before the performance test was conducted.

### 2.4. Testing and Characterization

The thermal conductivity of rubber composites was tested with a thermal conductivity meter (TC3200, Xi’an Xiaxi Electronic Technology, Xi’an, China) in accordance with ISO 22007-2008 standard [22]. The final results of the above tests are the average of five measurements.

The tensile and tear strength of rubber composites were tested using a tensile tester (5969, Instron, Norwood, MA, USA) in accordance with the GB/T 528-2009 standard. The preparation direction of the dumbbell specimen was along the calendering direction, and the tensile rate was 500 mm/min. The tear specimen was a standard right-angle specimen, and the testing sample preparation direction was along the rolling direction.

The fractured samples of SBR composites were observed using scanning electron microscopy (GeminiSEM 360, ZEISS, Jena, Germany) to examine their microscopic morphology. The samples were fixed onto the sample stage with conductive adhesive and were pre-coated with gold, using a gold sprayer.

In order to determine the molecular structure of GF before and after modification, Fourier-transform infrared spectroscopy analysis (FT-IR) was carried out using a Fourier-transform infrared spectrometer (INVENIO-S, Bruker, Mannheim, Germany). GF and MGF were prepared by the standard KBr compression method. The scanning range was 4000–400 cm^−1^ and the resolution was 4 cm^−1^. Each sample was scanned three times under the same conditions.

In order to quantitatively characterize the grafting rate of MGF, cut the GF and MGF samples after drying, weigh about 5 mg of each sample, and measure them in the thermogravimetric analyzer (TGA/DSC1/1600, METTLER TOLEDO, Columbus, OH, USA). The test temperature starts at 30 °C; control the heating rate of 10 °C/min, and raise it to 900 °C under a flow rate of 100 mL/min N_2_. For the TESPT samples after drying, weigh about 5 mg of each sample, and measure them in the thermogravimetric analyzer; the test temperature starts at 30 °C. Control the heating rate of 10 °C/min, and raise to 300 °C under a flow rate of 100 mL/min N_2_.

To test the vulcanization characteristics of the rubber compound, a rotorless vulcanizer (MDR 3000 Basic, MonTech, Germany) was used to obtain the vulcanization curve. The vulcanization curve provided information about the entire process of vulcanization, including the minimum torque (ML), maximum torque (MH), positive curing time, and other curing characteristics.

To investigate the rheological behavior of the unvulcanized rubber composite, a rubber processing analyzer (RPA 3000, MonTech, Germany) was utilized. Strain amplitude (γ) sweep from γ = 0.01° to 10° at 1 Hz, and frequency (*f*) sweep from *f* = 0.01 to 50 Hz at γ = 1° were conducted.

Additionally, the dynamic mechanical properties of the vulcanized composite were studied using a dynamic thermomechanical analyzer (DMA 242 E Artemis, NETZSCH, Selb, Germany) under the following test conditions: double cantilever mode, frequency of 1 Hz, amplitude of 10 μm, temperature sweep range from -100 °C to 80 °C, and a heating rate of 5 °C/min.

## 3. Results

### 3.1. GF Surface Modification

**Figure 1:**
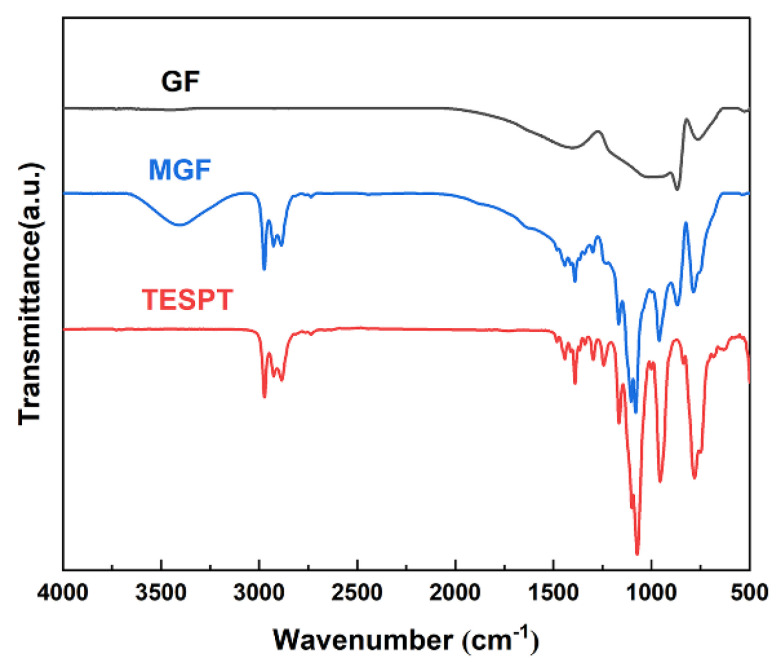
FT-IR infrared spectra of TESPT, MGF and GF.

The FT-IR spectra of GF and MGF are depicted in Figure 1. Notably, MGF exhibits newly added stretching vibration peaks of -CH- at 2975 cm^−1^, Si-O-C stretching vibration peaks at 1168 cm^−1^ and 1076 cm^−1^, a Si-O-Si stretching vibration peak at 958 cm^−1^, and a C-S stretching vibration peak at 787 cm^−1^, compared to pristine GF fibers. These observations suggest that the modification process yields more active functional groups on the fiber surface. The peak observed at approximately 3300 cm^−1^ in MGF is attributed to the -OH group. Additionally, the intensity of peaks at 813 cm^−1^ due to the Si-CH_3_ group decreases. These changes could be attributed to the efficient hydrolysis of the silane coupling agent under this experimental condition.

**Figure 2:**
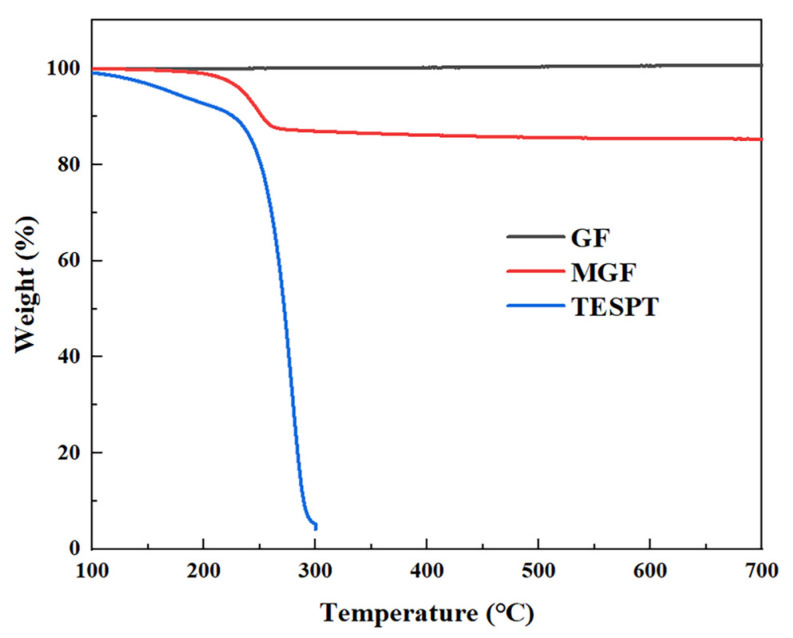
TGA curves of TESPT, MGF and GF.

TGA analysis was conducted to quantitatively characterize the grafting rate of MGF samples. Figure 2 displays the TGA curves of the GF, TESPT and MGF samples. GF exhibits no significant weight loss phenomenon. On the other hand, TESPT experiences complete degradation at temperatures ranging from 200 °C to 300 °C. However, MGF, after undergoing surface modification treatment, exhibits a notable thermal degradation stage (200~280 °C). This stage mainly involves the decomposition of TESPT grafted onto the fiber surface, including the decomposition of some methyl groups and various oxygen-containing functional groups. The weight loss rate for the decomposition of these groups is 12.81%, indicating a TESPT surface modification rate of 12.81%.

### 3.2. Thermal Conductivity of SBR/SA Composites

**Figure 3:**
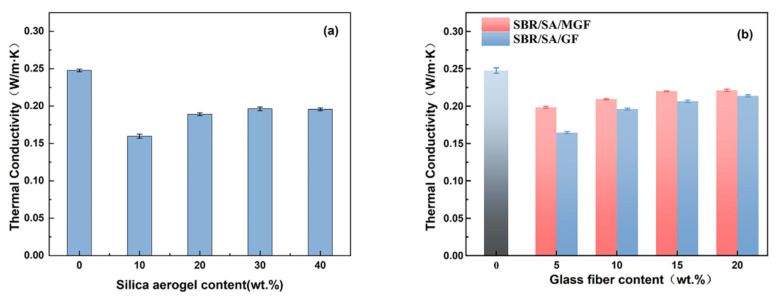
Thermal conductivity of SBR/SA/GF (**a**) and SBR/SA/MGF composites (**b**).

Figure 3 displays the thermal conductivity of rubber composites containing SBR/SA, SBR/SA/GF, and SBR/SA/MGF. It can be observed that the thermal conductivity of pure SBR rubber is 0.2476 W/(m·K). When the mass of SA is 10 wt% of SBR, the thermal conductivity (*λ*) is 0.1597 W/(m·K). Halim et al. [23] demonstrated that the addition of 9 wt% SA to silicone rubber (SiR) reduced the λ value by 0.18 W/(m·K). Fini Bidgoli et al. [24] added the same mass fraction of nano-SiO_2_ and SA into a nitrile-butadiene rubber (NBR) matrix and found that the thermal conductivity of the composites decreased by 15% and 43%, respectively, due to the unique nanoscale porous structure of SA. This structure can effectively reduce heat conduction through the solid, and each pore wall acts as a heat shield, blocking heat transfer and improving the thermal insulation performance of the composite material. Dispersibility also has a significant impact on the thermal conductivity of aerogel composites [25]. When the volume fraction of aerogel in the composite reaches a certain value, the aerogel particles aggregate, resulting in a clustered state [26]. The clustered state is known to be beneficial to thermal conductivity; thus, the thermal conductivity of composites increases with further increasing SA content. Hence, the results indicate that the addition of 10 wt% SA to the SBR composite yields the best thermal insulation performance. For the purposes of this study, the SA content remains constant at 10%, while the compounding ratio of GF is varied. The obtained SBR/SA/GF and SBR/SA/MGF rubber composites with varying contents’ thermal conductivity are shown in Figure 3b. The thermal conductivity of the composite is slightly increased but still significantly lower than that of pure SBR (0.2476 W/m·K). This indicates that the addition of GF to the composites promotes thermal network formation, which becomes more apparent with increasing fiber content [27,28,29]. However, it is worth noting that the thermal conductivity of the SBR/SA/MGF composite increases, compared to the addition of the same mass fraction of SBR/SA/GF [30]. The primary reason for this phenomenon is that the modified MGF has a good bridging effect with the matrix, which can reduce the void volume in the composite system, improve the contact surface between the fiber and the rubber, reduce the interfacial thermal resistance of the material, and establish a more comprehensive thermal conduction pathway.

### 3.3. Rheological Behavior of Unvulcanized SBR/SA/GF and SBR/SA/MGF Composites

Understanding the viscoelastic properties of rubber composites during processing and in final products requires rheological studies prior to vulcanization. In this study, we used the RPA3000 rubber processing analyzer to investigate the changes in storage modulus (*G*′) and loss modulus (*G*″) of various SBR composite materials with strain and frequency.

**Figure 4:**
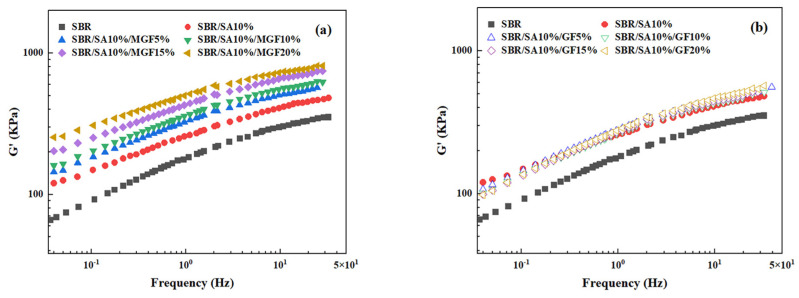
*G*′ as a function of *f* for SBR/SA/MGF (**a**), and SBR/SA/GF rubber composites (**b**).

**Figure 5:**
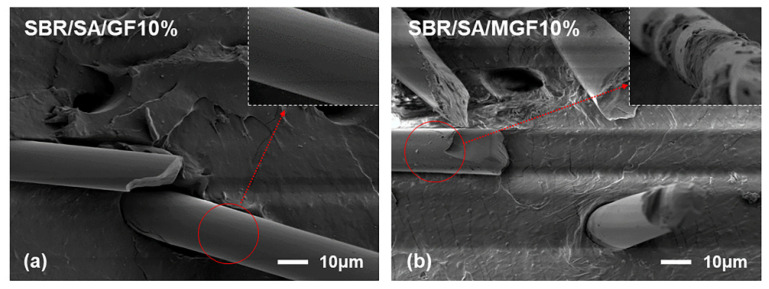
SEM images of SBR/SA/GF10% (**a**) and SBR/SA/MGF10% (**b**).

Figure 4 displays the frequency-dependent dynamic moduli curves of rubber composites. The decreasing slope of lg *G*′~lg *ω* indicates a correlation between the introduction of MGF and the long-term relaxation unit in the system, with the slope of the logarithmic coordinate curve of *G*′~*ω* decreasing from 3.07 to 2.37 in the low-frequency region, suggesting the influence of MGF. Conversely, GF seems to have little impact on the G′~*ω* relationship. The surface-active groups of MGF can encapsulate and immobilize macromolecules to form more bound rubber with restricted relaxation, explaining the observed behavior [31,32,33]. Further evidence was obtained in Figure 5 through SEM images of the surfaces of MGF and GF in rubber composites. A layer of SBR rubber matrix covered the surface of modified MGF, while the surface of GF was smooth, without obvious attachments. This confirms that the modified MGF and SBR rubber form a firm bond, and the surface-active groups can adsorb more rubber matrix for enhanced properties.

**Figure 6:**
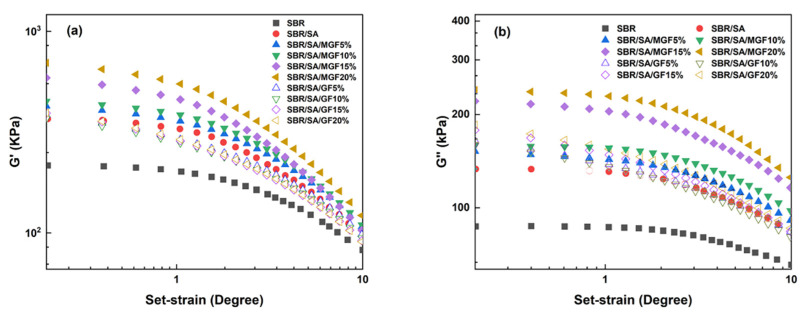
*G*′ (**a**) and *G*″ (**b**) as a function of *γ* for SBR rubber composites.

**Figure 7:**
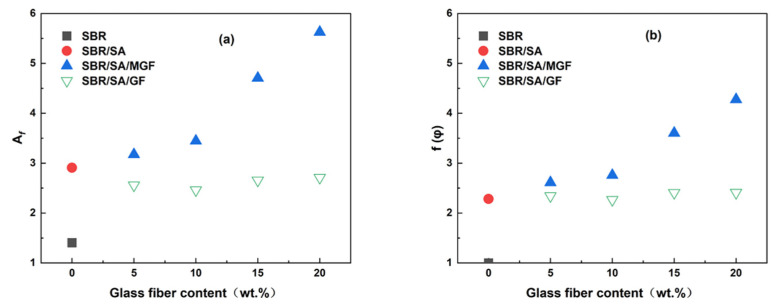
The $A_{f}\left(\varphi \right)$ (**a**) and $f\left(\varphi \right)$ (**b**) as a function of glass fiber contents.

Figure 6 illustrates *G*′ and *G*″ as a function of strain in the uncured state. SA exhibits a substantial additional reinforcement effect at lower strains. Upon the addition of fibers, the *G*′ of SBR/SA/MGF is observed to be higher than that of SBR/SA/GF [34]. Upon macroscopic strain application to the composite material, a strain amplification effect arises [35,36]. Based on the strain amplification factor and “particle phase” structural effects, Song and Zheng proposed the “two-phase model” [37,38,39]. This model decomposes the rheological response of the composite system into the sum of independent contributions of the polymer matrix and filler particles to the viscoelasticity, specifically expressed as

$$G^{*}\left(\varphi ,\omega \right)=A_{f}\left(\varphi \right)G_{m}^{*}\left(\omega \right)+G_{f}^{*}\left(\varphi ,\omega \right),$$

where $G_{m}^{*}$ represents the matrix modulus, $G_{f}^{*}$ represents the filler phase modulus, and $A_{f}\left(\varphi \right)$ represents the strain amplification factor. In this study, $G_{0}^{\prime }\left(\varphi \right)$ is used to represent the linear modulus of the composite material, and $G_{0}^{\prime }m$ represents the linear modulus of the matrix. The linear viscoelastic region reinforcement factor

$$f\left(\varphi \right)=G_{0}^{\prime }\left(\varphi \right)/G_{0}^{\prime }m$$

and the strain amplification factor $A_{f}\left(\varphi \right)$ change with the mass fraction of filler particles, as depicted in Figure 7a,b. When the content of GF is the same, the $f\left(\varphi \right)$ and $A_{f}$ of the SBR/SA/MGF compound are higher than those of the SBR/SA/GF compound, indicating that the reinforcing effect of MGF is better than that of GF [40,41].

**Figure 8:**
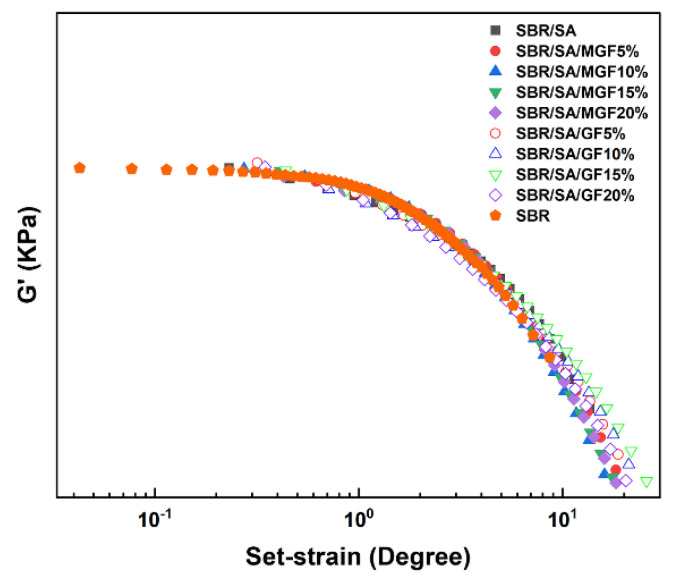
The vertical and horizontal translation of *G*′-*γ* relation curve.

These curves demonstrate the typical nonlinear relationship of modulus increasing with strain, commonly known as the “Payne effect”. The Payne effect is attributed to various mechanisms, including the agglomeration/deagglomeration of filler aggregates [42,43], crushing/reforming of the filler network [44,45] or polymer-filler network [42], and unwrapping of the adsorption chain [46]. Figure 8 shows that the *G*′-*γ* curve’s vertical and horizontal translation of the filling system can form a superimposed curve based on SBR. On the one hand, this result demonstrates that particles play a role in strain amplification and promote the disentanglement of the molecular chains of the SBR matrix [36]. On the other hand, it indicates that the nonlinear viscoelasticity is not related to assumptions such as the failure of filler particle agglomerates or the desorption of matrix molecular chains [47]. Simultaneous measurements of rheological response and electrical conductance of composites under large strain amplitudes by Yihu Song et al. [48] found that the destruction and recovery of carbon black (CB) agglomerates were not synchronized with the appearance of the Payne effect, indicating that the structural evolution of the filler phase was not the dominant factor in the Payne effect.

### 3.4. Vulcanization Characteristics of Rubber Composites

**Figure 9:**
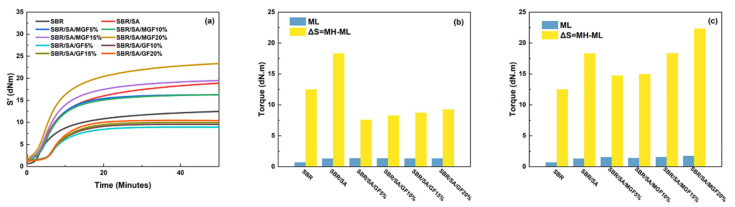
Vulcanization characteristic curves of rubber composites (**a**), ML and ΔS of SBR/SA/GF composites (**b**), and ML and ΔS of SBR/SA/MGF composites (**c**).

Figure 9a displays the vulcanization curve of the SBR rubber composite; the vulcanization characteristics including the ML, MH, and the difference between the maximum and minimum torque (ΔS) of the composite system are presented in Figure 9b,c. ML is typically associated with the viscosity of the rubber compounding system [49]. By comparing Figure 9b,c, it can be observed that the addition of SA, GF, and MGF leads to an increase in the viscosity of the composite system. Moreover, as more fibers are added to the SBR matrix, the composite system exhibits higher viscosity [50].

ΔS is often used to infer rubber and filler-rubber crosslink density [51]. Figure 9b shows that the crosslinking density of the SBR/SA/GF composite is reduced compared to that of the SBR/SA composite. Utara et al. [52] reported an increase in crosslink density when hemp fibers were added to the rubber matrix, attributed to the good interaction between the fibers and the matrix. On the other hand, Mujtaba et al. [53,54] reported the opposite, and they believed it to be the result of partial inactivation of the crosslinker due to the absorption on the large surface of the filler particles. We believe that the decrease in crosslink density may be due to the difficulty in dispersing the fiber, causing “macro” agglomeration (relative to SA) to form a barrier that hinders the diffusion of the vulcanizing agent, thereby reducing the crosslink density. However, as shown in Figure 9b, the modified MGF increases the crosslinking density of the SBR/SA/MGF composite system. This is attributed to the introduction of numerous active groups on the surface, which may hinder the diffusion of vulcanizing agents. Nevertheless, the presence of TESPT on the surface of MGF participates in the vulcanization reaction, ultimately enhancing ΔS [55].

### 3.5. Dynamic Mechanical Properties of Vulcanized Composites

**Figure 10:**
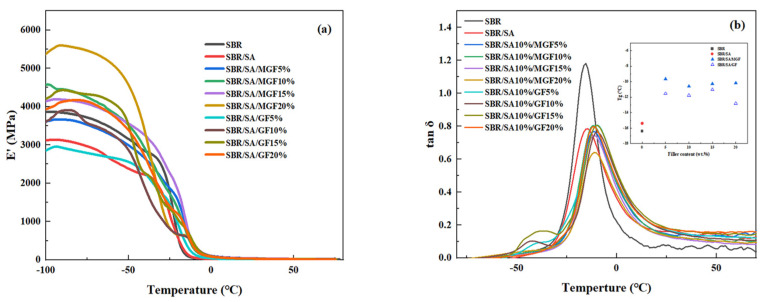
Dynamic mechanical properties of SBR/SA/GF, SBR/SA/MGF composites, *E*′~T (**a**) and tan*δ*~T (**b**).

Figure 10 shows the temperature dependence of the storage modulus (*E*′) and loss factor (tan*δ*) of the rubber. It can be observed that *E*′ decreases with increasing temperature. Moreover, the low-temperature storage modulus of the rubber increases first and then decreases as the GF content increases, reaching a maximum value at 15 wt%. The fact that the *E*′ value in the glassy state (at −60 °C) slightly increases with the fiber amount indicates a good dispersion of the reinforcement particles. However, at higher fiber content (20 wt%), the fibers could not achieve good dispersion, leading to a reduced reinforcing effect and decreased storage modulus. When comparing SBR/SA/GF to SBR/SA/MGF with the same fiber content, it is found that SBR/SA/MGF has a higher *E*′ value. This is because TESPT participates in the vulcanization and cross-linking of the rubber, forming a reinforcing network.

The *T_g_* of the vulcanizate after adding MGF is higher than that of the SBR/SA vulcanizate, indicating that the movement of the SBR molecular chain is constrained. This suggests that the modified GF has a close association with the rubber molecular chain, and the interfacial interaction force between them is enhanced.

It has been shown that the relationship between *M*_c_ and the shear storage modulus *E*′ follows this Equation [56]:

$$M_{C}=3\cdot \frac{\rho RT}{E^{\prime }}$$

In this way, the molecular weight between cross-links and cross-link density [57] can be measured through use of the DMA.

**Figure 11:**
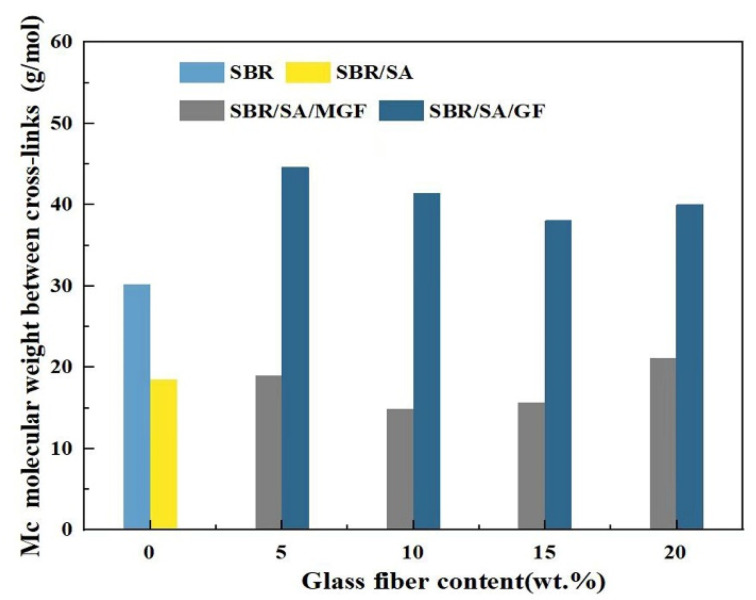
The molecular weight between the crosslinking points of rubbers calculated by DMA.

As seen from Figure 11, the *M*_c_ of SBR/SA/MGF specimens reduced from 21 to 15 g/mol when the content of MGF content increased to 10 wt%, meaning the crosslinking degree of the resulting products increases in an obvious way, which is consistent with the results obtained from vulcanization characteristics.

### 3.6. Mechanical Properties of SBR/SA/GF and SBR/SA/MGF Composites

**Figure 12:**
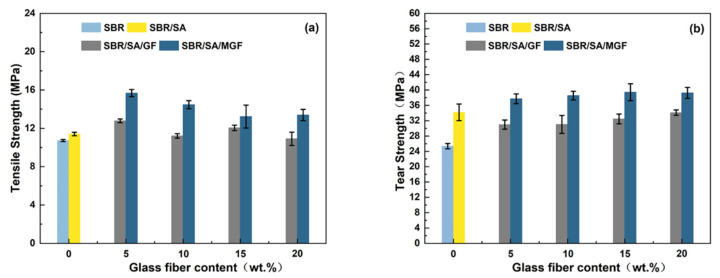
Tensile strength (**a**) and tear strength (**b**) of SBR/SA/GF and SBR/SA/MGF composites.

Figure 12a shows a trend of increasing and then decreasing tensile strength for the composites before and after GF-fiber modification [58]. The mechanical properties of short fiber/rubber composites depend on several factors, including dispersion level, fiber orientation, and interfacial strength of the composite. The higher tensile strength of the SBR/SA/MGF composites under the same fiber loading can be mainly attributed to the presence of the silane coupling agent TESPT, which has active groups such as polysulfide bonds, Si-O bonds, and Si-C bonds. The polysulfide bonds participate in the rubber vulcanization process, forming a chemical cross-linking bond with the rubber matrix, enhancing the interfacial interaction force between the fiber and the rubber matrix. This enables effective stress transfer from the SBR to the MGF through the interface and improves the composite material’s mechanical properties [55]. The tensile strength of the SBR/SA/GF system increased by 5~12% compared to SBR/SA, while the SBR/SA/MGF composite’s tensile strength increased by 15~37%, indicating a significant reinforcing effect of the MGF/SA composite material when used in combination.

Figure 12b illustrates the positive effect of increasing MGF content on tear strength in the composites. The addition of surface-modified fibers effectively hinders the propagation of material cracks [34]. When the crack expands and encounters the fiber, it changes the original expansion direction and may bypass the nearby matrix or interface layer or even develop along the orientation direction of the fiber. This redirection improves tear strength, leading to a 10-15% increase in the tear strength of the SBR/SA/MGF system compared to SBR/SA. Conversely, the tear strength of the SBR/SA/GF composite system decreases due to fiber entanglement caused by the smooth GF surface, leading to fiber debonding and accelerated composite fracture.

**Figure 13:**
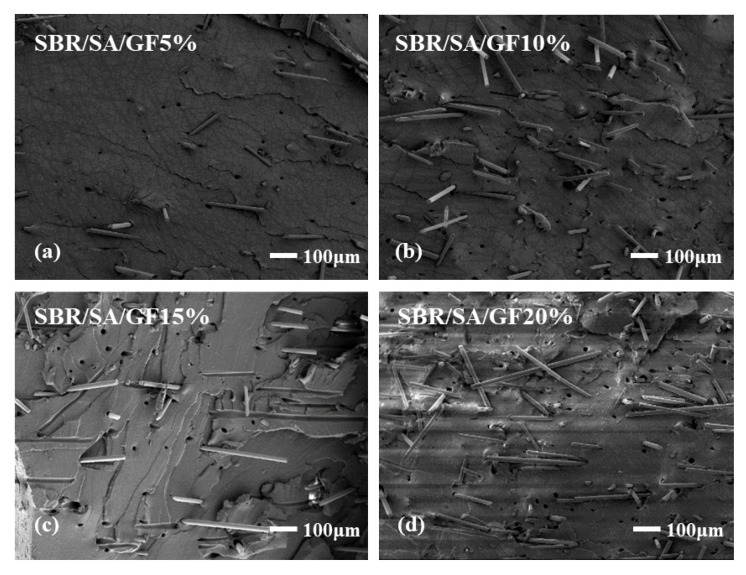

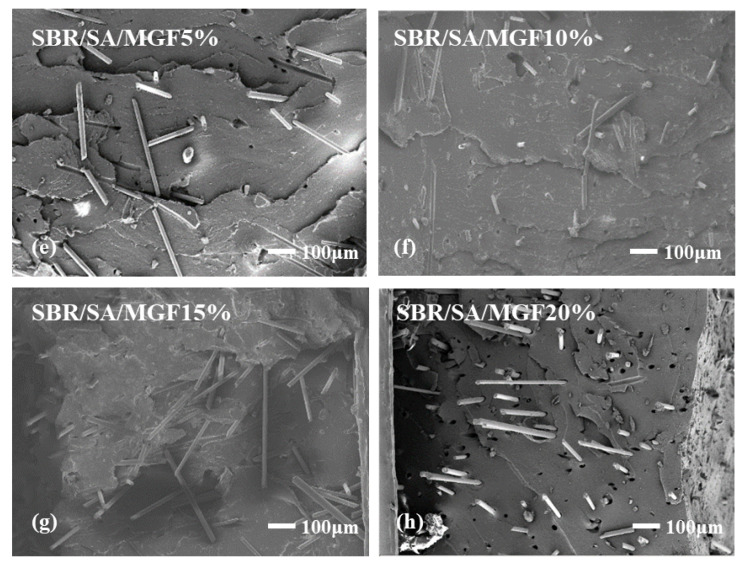
SEM images of SBR/SA/GF5% (**a**), SBR/SA/GF10% (**b**), SBR/SA/GF15% (**c**), SBR/SA/GF20% (**d**), and SBR/SA/MGF5% (**e**), SBR/SA/MGF10% (**f**), SBR/SA/MGF15% (**g**), SBR/SA/MGF20% (**h**).

To examine the interface state of the composite rubber material, the tensile section’s microscopic morphology was observed by SEM, as presented in Figure 13. It is observed that the fibers on the tensile fracture surface of the SBR/SA/GF composite are arranged in a disorderly way, and some fibers are pulled out on the fracture surface. 

Table 2 shows the fiber extraction rate analyzed using ImageJ software, with an increase in GF content; defects such as holes and cracks in the composite material also increase, and the fiber extraction rate rises. For SBR/SA/MGF composites, the composite material’s extraction rate decreases significantly compared to SBR/SA/GF, as more active groups appear on the surface of the modified GF fiber, enhancing its affinity with the rubber matrix and strengthening the interface bonding with the SBR rubber.

## 4. Conclusions

With the addition of SA, the thermal conductivity of SBR is reduced by 35% owing to the heat shield of the nanoscale porous structure of SA. Furthermore, when MGF (modified glass fibers) are added to the SBR/SA composite, there is a noticeable improvement in tear strength of approximately 10% to 15% and tensile strength of 15% to 37%. In the SBR/SA/MGF composite, the surface of MGF is rich in active groups due to the modification with the silane coupling agent TESPT. The polysulfide bond within MGF participates in the rubber vulcanization process, strengthening the interfacial adhesion with the rubber matrix and significantly improving the crosslinking density and mechanical properties of the composite material. Moreover, the chopped fibers after surface modification of MGF induce a more perfect filler network structure; this enhanced filler network structure contributes to the reinforcement of the composite and aggravates the Payne effect, which amplifies the strain-amplifying behavior of the composite system.

## Figures and Tables

**Table 1:** Composition of SBR composites.

Samples	SA/SBR (wt%)	GF/SBR (wt%)	MGF/SBR (wt%)
SBR/SA10%	10.0	0	0
SBR/SA20%	20.0	0	0
SBR/SA30%	30.0	0	0
SBR/SA40%	40.0	0	0
SBR/SA/GF5%	10.0	5.0	0
SBR/SA/GF10%	10.0	10.0	0
SBR/SA/GF15%	10.0	15.0	0
SBR/SA/GF20%	10.0	20.0	0
SBR/SA/MGF5%	10.0	0	5.0
SBR/SA/MGF10%	10.0	0	10.0
SBR/SA/MGF15%	10.0	0	15.0
SBR/SA/MGF20%	10.0	0	20.0

**Table 2:** The porosity of the fracture surface of various composite rubbers.

Samples	Porosity of Fracture Surface (%)
SBR/SA/GF5%	0.188
SBR/SA/GF10%	0.622
SBR/SA/GF15%	1.032
SBR/SA/GF20%	1.468
SBR/SA/MGF5%	0.14
SBR/SA/MGF10%	0.091
SBR/SA/MGF15%	0.474
SBR/SA/MGF20%	0.647

## Data Availability

The data that support the findings of this work are available from the corresponding author on reasonable request.
